# Effective Material Basis and Mechanism Analysis of Compound Banmao Capsule against Tumors Using Integrative Network Pharmacology and Molecular Docking

**DOI:** 10.1155/2021/6653460

**Published:** 2021-05-04

**Authors:** Tian-Mu He, Jing-Xian Liu, Can-Can Duan, Xiao-Fei Li, Jian-Yong Zhang

**Affiliations:** ^1^Basic Medicine School, Zunyi Medical University, Zunyi, China; ^2^School of Pharmacy, Zunyi Medical University, Zunyi, China; ^3^Key Laboratory of Basic Pharmacology Ministry Education and Joint International Research Laboratory of Ethnomedicine Ministry of Education, Zunyi Medical University, Zunyi, China

## Abstract

**Purpose:**

Compound banmao capsule (CBC), a well-known traditional Chinese medical material, is known to inhibit various tumors. However, its material basis and pharmacological mechanisms remain to be elucidated. This study aimed to investigate the effective material basis and mechanisms of action of CBC against tumors.

**Methods:**

Active compounds of CBC were identified using public database and reports to build a network. The corresponding targets of active compounds were retrieved from online databases, and the antitumor targets were identified by GeneCards database. The antitumor hub targets were generated via protein-protein interaction analysis using String, and key compounds and targets from the integrative network were detected by molecular docking and ADMET. Top targets in hepatocellular carcinoma were confirmed by quantitative real-time PCR (qPCR). Finally, the multivariate biological network was built to identify the integrating mechanisms of action of CBC against tumor cells.

**Results:**

A total of 128 compounds and 436 targets of CBC were identified successfully. Based on the generated multivariate biological network analysis, 25 key compounds, nine hub targets, and two pathways were further explored. Effective material bases of cantharidin, baicalein, scutellarin, sesamin, and quercetin were verified by integrative network analysis. PTGS2, ESR1, and TP53 were identified as hub targets via multivariate biological network analysis and confirmed using qPCR. Furthermore, VEGF and estrogen signaling pathways seem to play a role in the antitumor activity of CBC. Thus, breast cancer may be a potential clinical indication of CBC.

**Conclusion:**

This study successfully identified the material basis of CBC and its synergistic mechanisms of action against tumor cells.

## 1. Introduction

Studies have shown that the five-year survival rate of cancer generally rise when tumor survival trends in global areas are evaluated. However, the survival rate of malignant tumors, such as liver cancer, is still low [1]. At present, antitumor therapy is mainly based on radiotherapy and chemotherapy, which are linked to severe adverse reactions [2]. Thus, exploring new antitumor drugs with fewer side effects is urgently needed. Traditional Chinese medicine (TCM) is popular, plays a role in the modern healthcare system, and can provide potential effective strategies towards the treatment of tumors [3]. Compared with conventional drugs, herbal drugs have been considered an important supplementary treatment to alleviate the side effects of both radiotherapy and chemotherapy [4].

Compound banmao capsule (CBC) is one of the most common antitumor TCM compounds composed of 11 herbs according to the Pharmacopoeia of the People's Republic of China (2020 edition), including the Mylabris (BM), Ginseng Radix Et Rhizoma (RS), Astragali Radix (HQ), Scutellariae Barbatae Herba (BZL), Curcumae Rhizoma (EZ), Acanthopanacis Senticosi Radix Et Rhizoma Seu Caulis (CWJ), Fel Ursi (XD), Glycyrrhizae Radix Et Rhizoma (GC), Corni Fructus (SZY), Ligustri Lucidi Fructus (NZZ), and Sparganii Rhizoma (SL). CBC has been used to clinically treat primary liver tumor, lung tumor, rectal tumor, and malignant lymphoma [5]. Most notably, it differs from other antitumor drugs as CBC not only can directly inhibit tumor proliferation but also enhance immunity of patients. Modern antitumor mechanisms of CBC mainly involve proapoptosis, protein phosphorylation, and immunoregulation [6–8]. However, the specific pharmacodynamic material and molecular mechanisms underlying tumor treatment of CBC still remain unclear.

Regarding the integrative network pharmacology, a multicompound, multitarget, and multipathway approach has been used in TCM, especially in antitumor research [9, 10]. Here, we attempt to identify effective material basis and molecular mechanisms of CBC using network pharmacology and molecular docking. Hub targets aim to be verified by quantitative real-time PCR (qPCR) in human hepatocellular carcinoma cells (SMMC-7721 cell), providing a theoretical basis for further research.

## 2. Materials and Methods

### 2.1. Network Pharmacology-Based Analysis

#### 2.1.1. Identification of Candidate Compounds of CBC

All compounds of 11 TCM herbs of CBC were retrieved from the encyclopedia of TCM (ETCM) database (http://www.nrc.ac.cn:9090/ETCM/) and literature reports. ETCM is an encyclopedia of TCM, which includes comprehensive and standardized information for the commonly used herbs and formulas of TCM as well as their ingredients [11].

#### 2.1.2. Screening for Bioactive Compounds of CBC

A quantitative metrics, known as the quantitative estimate of drug likeness (QED), was used to assess drug likeness. Estimated values ranged from 0 (all properties are unfavorable) to 1 (all properties are favorable) [12]. ETCM classified all 7274 ingredients into three groups according to their QED scores: good (QED > 0.67), moderate (0.49 ≤ QED ≤ 0.67), and weak (QED < 0.49) [11]. Compounds of CBC evaluated as good (QED > 0.67) were collected as bioactive compounds. Likewise, active compounds that did not fulfill the criteria for QED but have been reported in the available literature were recruited separately.

#### 2.1.3. Prediction of Drug Targets for CBC and Network Construction

Targets of active compounds of CBC were retrieved from ETCM. Other active compounds not included in ETCM were drawn via ChemDraw 19.0 software and imported into TargetNet database (http://targetnet.scbdd.com/), a web service for predicting potential drug-target interaction profiling via multitarget SAR models, for predicting targets [13]. Targets were filtered by accuracy (AUC) ≥ 0.7 and probability (Prob) ≥ 0.9. Targets were corrected and transformed using UniProt database (https://www.uniprot.org/) and defined as *Homo sapiens*. Subsequently, correction targets were imported into Cytoscape 3.7.0 software to construct a network of herb-compounds-targets.

#### 2.1.4. Collection of Gene Targets Associated with Tumors

The terms “anti-tumor” and “anti-cancer” were used as search terms in the GeneCards database (https://www.genecards.org/) [14], a searchable and integrative database that provides comprehensive and user-friendly information on all annotated and predicted human genes. The targets generated by the intersection between the tumor-related targets and predicted targets of CBC were identified as tumor-targets of CBC.

#### 2.1.5. A Protein-Protein Interaction (PPI) Network Construction and Hub Targets Screening

The tumor-targets of CBC were submitted to STRING 11.0 database (https://string-db.org/) [15], which stores information about protein interactions. Only proteins with the confidence score higher than 0.9 were selected. The protein information was imported into Cytoscape to construct the protein-protein interaction (PPI) network and merged with the herb-compounds-targets network. Finally, the hub targets and core compounds were screened by the values of degree and betweenness for constructing the core compound-target network. The hub targets were applied for enrichment and antitumor location analysis.

#### 2.1.6. Network Construction of Nature and Flavor, Channel Tropism, and Tumor Location of CBC

Herbal drug properties in CBC were extracted from the Pharmacopoeia of the People's Republic of China according to the Four Properties (warm, cold, hot, and cool) and Five Flavors (pungent, sweet, sour, bitter, and salty) [16]. The nature and flavor (Chinese name, Xing wei), including Four Properties and Five Flavors, also called drug properties in herbal drug, which were clinically used according to clinical treatment experience of presented clinical symptom in patients in TCM theories. The channel tropism (Chinese name, Gui jing) is a theory of positioning and orientation of a certain organ, meridian, or specific clinical indication location to reveal the effect of TCM.

The nature and flavour and channel tropism of CBC were collected to construct a network via Cytoscape to analyze drug properties and clinical indications. Meanwhile, hub targets filtered by PPI network were uploaded to the Comparative Toxicogenomics Database (http://ctdbase.org/) to collect diseases' information. Diseases marked with “marker/mechanism” and defined as cancer were selected and imported into Cytoscape to explore the cluster site of CBC clinical indication.

#### 2.1.7. Gene Ontology and Pathway Enrichment Analysis of CBC

To evaluate the role of targets by bioinformatic annotation, hub targets were imported into Enrichr database (http://amp.pharm.mssm.edu/Enrichr/) [17], which currently contains a large collection of diverse gene set libraries available for analysis. Hub targets were utilized by Enrichr high-throughput functional annotation bioinformatics to perform functional annotation, enrichment analysis, and gene ontology (GO) terms based on Kyoto Encyclopedia of Genes and Genomes (KEGG) pathways, with *P* < 0.05. Terms and pathways were sorted based on combined scores, and target-pathway network was constructed via Cytoscape to display the tightly connection between targets and tumor-related pathways.

#### 2.1.8. Integrative Mechanism Analysis of CBC in Tumor Treatment

To further explore potential mechanisms of CBC against tumor, networks of the core compound-target and target-pathway were merged to construct the herb-compound-target-pathway integrative network. The major tumor pathways were screened; among them, linked compounds and targets were identified as key compounds and targets subsequently. The integrative network was further verification of key compounds and targets, to integrate characteristics of multicompound, multitarget, and multipathway of CBC. Finally, the multivariate biological network was merged with the herb-compound-target-pathway, drug properties, and antitumor location network for comprehensive integration.

#### 2.1.9. ADMET Absorption Level Analysis and Molecular Docking Simulation

Effective material basis of CBC was verified by ADMET absorption level and molecular docking. ADMET absorption level of compounds was obtained using the ETCM database. Levels of “0, 1, 2, and 3” meant “good, moderate, low, and very low,” respectively. Key compounds with good ADMET absorption levels were selected for the integrative network analysis [18].

Molecular docking was performed to simulate structural stability between key compounds and targets using iGEMDOCK 2.1 software, a commonly used tool for molecular recognition, which evaluates and improves scoring functions [19]. The iGEMDOCK energy functions consist of electrostatic, steric, and hydrogen bonding potentials, which were used to recognize complexes. The key compounds in herb-compound-target-pathway network were downloaded using PubChem database (https://pubchem.ncbi.nlm.nih.gov/), energy minimized via Chem3D 19.0 software, and saved in MOL format. Protein targets were downloaded from PDB database (http://www.rcsb.org/). The standard docking settings were selected via iGEMDOCK: population size = 200; generations = 70; number of solutions = 2. Results showed that lower energies correlated with more stable constructions. Finally, effective material basis of CBC was identified by ADMET absorption level analysis and molecular docking.

### 2.2. Experimental Validation

#### 2.2.1. Cell Culture

Human SMMC-7721 cells (ATCC, USA) were chosen to validate our experiments. Cells were cultured in RPMI 1640 medium (Gibco, USA) supplemented with 10% FBS (Gibco, USA), 100 U/ml penicillin, and 100 mg/ml streptomycin (Solarbio, China) and maintained at 37°C in a humidified chamber under 5% CO_2_.

#### 2.2.2. Quantitative Real-Time PCR Analysis

The hub targets with higher degree, linked to hepatocellular carcinoma in the antitumor location network, were verified by qPCR. The SMMC-7721 cells (4 × 10^6^ cells) were seeded in 100 mm dishes and incubated for 24 h. After, pretreatment with a range of CBC concentrations (Guizhou Yibai Pharmaceutical, China, Z52020238) (0, control; 0.01 mg/ml, LCBC; 1 mg/ml, HCBC) in 12 h for RNA extraction was completed. Total RNA separation and extraction were performed according to the instructions of the TaKaRa MiniBEST Universal RNA Extraction Kit (TaKaRa, Clontech). Reverse transcription reactions were performed using 900 ng of RNA with PrimerScript™ RT Master Mix (Perfect Real Time) for cDNA.  Table 1 lists the primer sequences. Gene expression of *PTGS2*, *TP53*, *ESR1*, *ABCB1,* and *TOP2A* was investigated. The samples were exposed to predenaturation at 95°C for 30 s, followed by 39 cycles of denaturation at 95°C for 5 s, and at 60°C for 30 s, and annealing at 95°C for 10 s. The dissolution curve conditions were 65°C for 0.05 s and 95°C for 0.5 s using 10 *μ*L TB Green Premix EX Taq II, 0.8 *μ*L 10 *μ*mol/L forward primer, 0.8 *μ*L 10 *μ*mol/L reverse primer, and 2 *μ*L cDNA. Water was added to achieve a total volume of 6 *μ*L. *GAPDH* was used as the internal control, and data were analyzed using the 2^−ΔΔCt^ method. The experiment was repeated three times.

### 2.3. Statistical Analysis

Statistical analysis was performed using Prism 8.0.2 software. Data were expressed as means ± SD and analyzed using Student's *t*-test. Differences between groups were considered to be statistically significant if *P* < 0.05.

## 3. Results

### 3.1. Network Pharmacology-Based Analysis

#### 3.1.1. Identification of Bioactive Compounds of CBC

**Figure 1:**
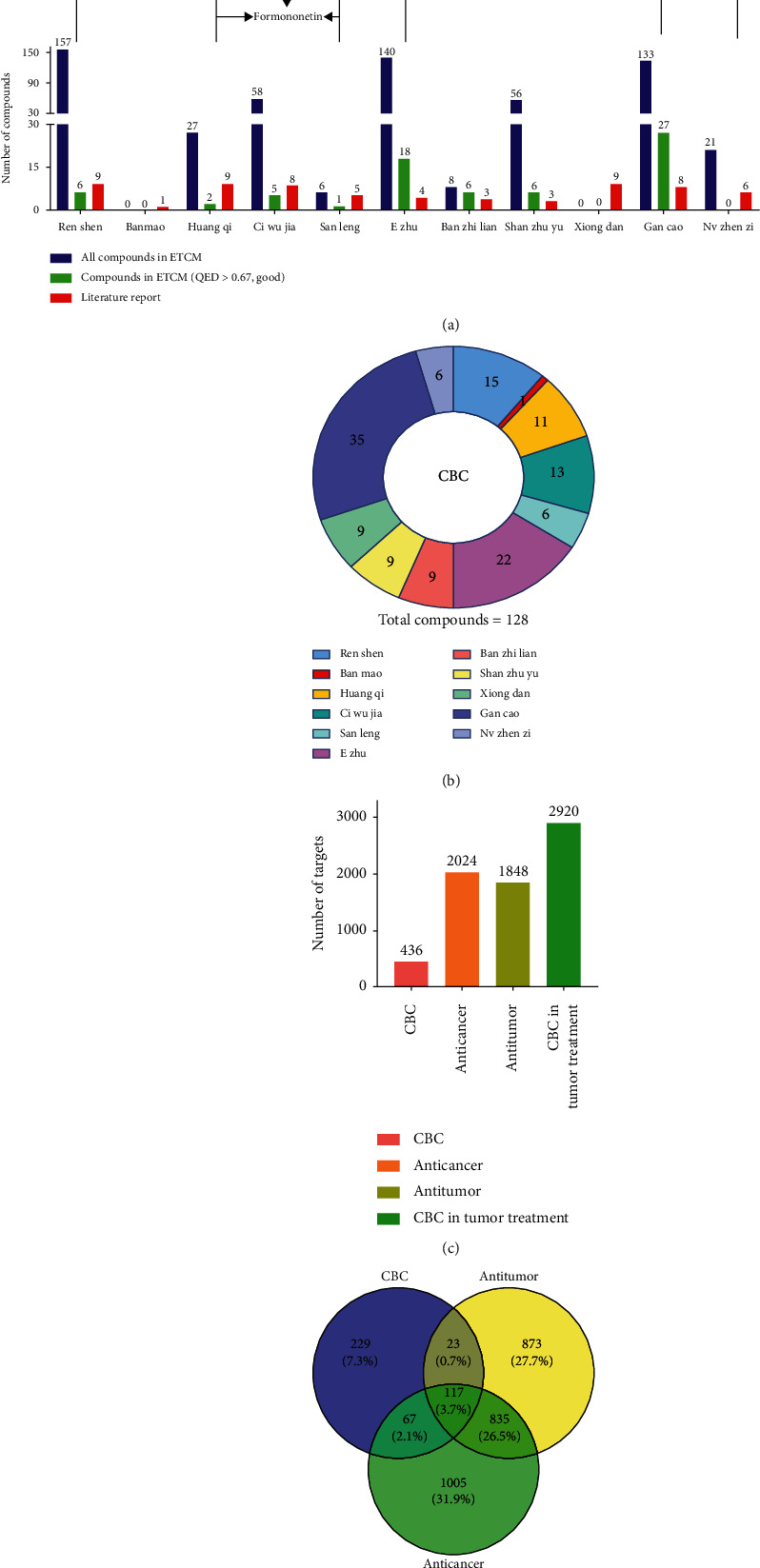
Diagram of compounds and targets in compound banmao capsule (CBC) against tumors. (a) Number of compounds of 11 herbs from CBC. (b) Proportion of compounds of each herb from CBC. (c) Number of targets in CBC and predicted tumor-targets. (d) Venn diagram of targets in CBC against tumors.

After deleting duplicate data, a total of 128 compounds were identified as candidate compounds (Table 2), including 69 compounds with good QED and 69 effective literature-based antitumor compounds (Figures 1(a) and 1(b)) [20–36]. Cantharidin (CTD), a major component in BM, presents a strong antitumor activity [26], and major quality markers including specnuezhenide, liquiritin, loganin, and syringin of CBC were also identified [35].

#### 3.1.2. Herb-Compound-Target Network Construction and Targets of CBC against Tumors

**Figure 2:**
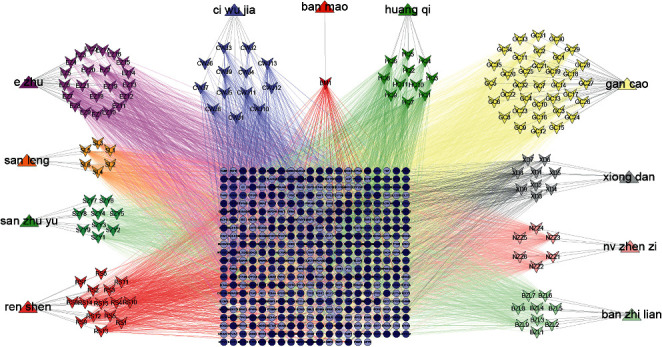
Herb-compound-targets network in compound banmao capsule (CBC). Triangle, quadrilateral, and round shapes stand for herb, compound, and target, respectively. Degree value changes with color and size variation.

**Figure 3:**
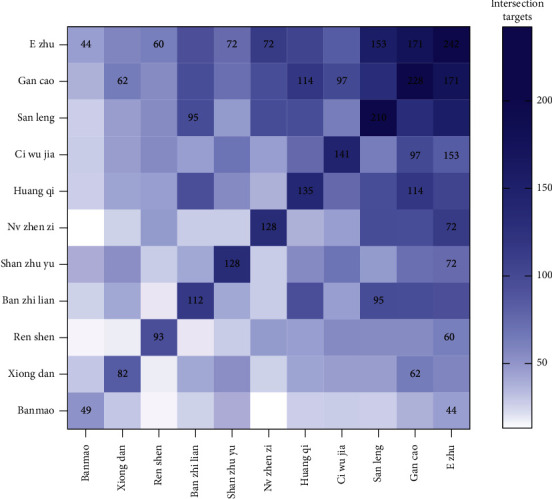
Intersection targets in each herb from compound banmao capsule (CBC). *X* axis and *Y* axis stand for herbs from CBC. The most intersection targets between two herbs from CBC were shown with number in the figure, and number of intersection target changes with color variation.

In this study, 436 targets were screened against compounds from CBC, which corresponded to an average of almost three targets per compound. The herb-compound-target network was constructed via Cytoscape software, where degree value changes with color and size variation (Figure 2). In order to identify correlation between each herb in CBC, a heat map was applied to reveal common number of targets. EZ correlated with 242 targets of CBC, while banmao was connected to 49 targets (Figure 3). Alternatively, by searching “anti-tumor” and “anti-cancer” in the GeneCards database, 1848 and 2024 targets were obtained, respectively. The 2920 common targets were selected (Figure 1(c)). Subsequently, the intersection of 207 CBC tumor targets was filtered out between disease targets and CBC targets (Figure 1(d)).

#### 3.1.3. PPI and Core Compound-Target Network Construction

**Figure 4:**
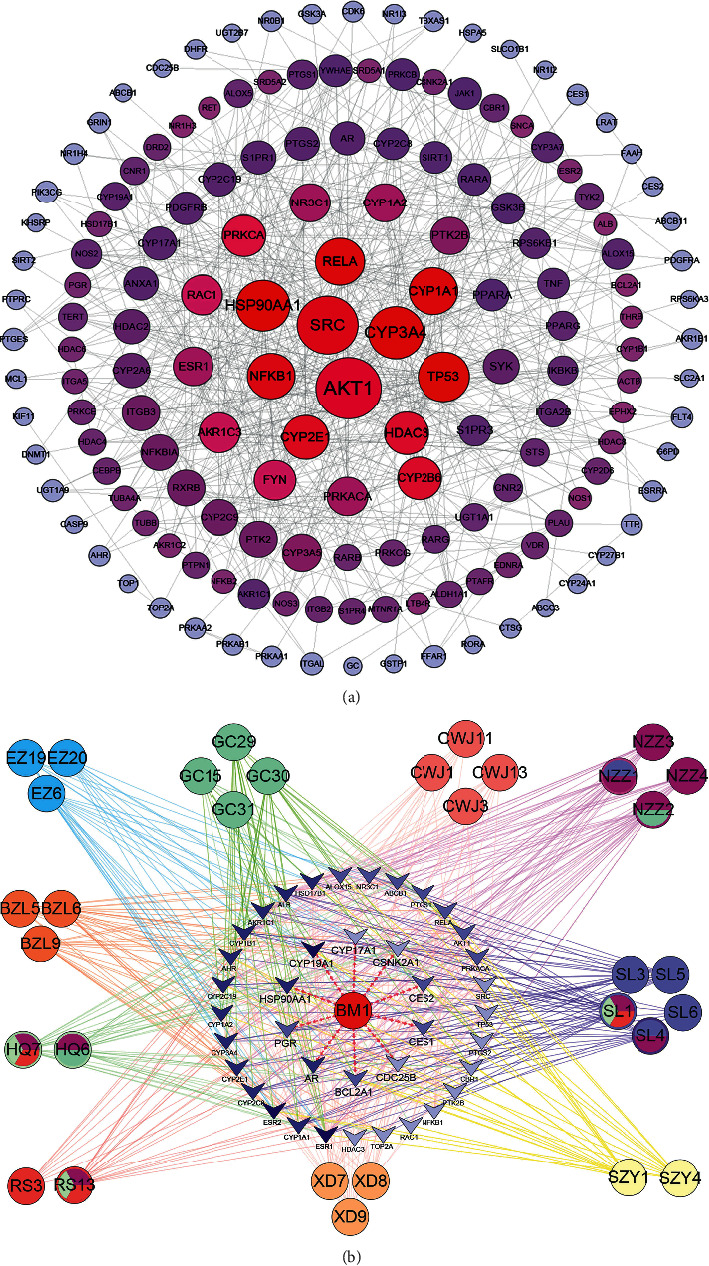
(a) Protein-protein interaction (PPI) network in compound banmao capsule (CBC) targets against tumors. Colors from blue to red and size change indicate that the degree value is increasing. (b) Core-compound-target network in CBC against tumors. Quadrilateral and round shapes stand for target and compound, respectively. Nodes and edges with the same color represent the same herb and related targets. Color from shallow to deep; the target degree value was increasing.

**Figure 5:**
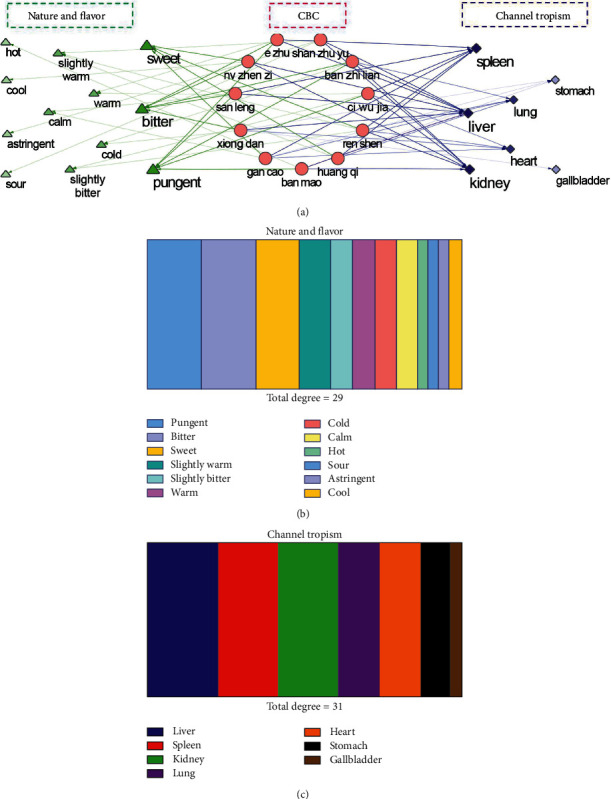
Nature and flavor and channel tropism in compound banmao capsule (CBC). (a) Detail mapping information of each herb from CBC. Green, red, and blue stand for nature and flavor, herbs from CBC, and channel tropism, respectively. (b) Detail degree value proportion in nature and flavor in herbs form CBC. (c) Detail degree value proportion in channel tropism in herbs form CBC.

Tumor-targets of CBC were brought to the String database to perform a PPI network with the highest confidence (0.9). A total of 158 nodes and 619 edges were obtained in the PPI network (Figure 4). The PPI network was merged with the herb-compounds-targets network to build the core compound-target network based on topological parameters with average degree value > 18.43 and average betweenness > 0.007. Then, 39 putative hub targets and 29 core compounds were identified (Figure 5), suggesting that the hub targets and compounds had multiple beneficial biological functions for treating tumors at the molecular level.

#### 3.1.4. Drug Properties and Organism Location of Antitumor Analysis

**Figure 6:**
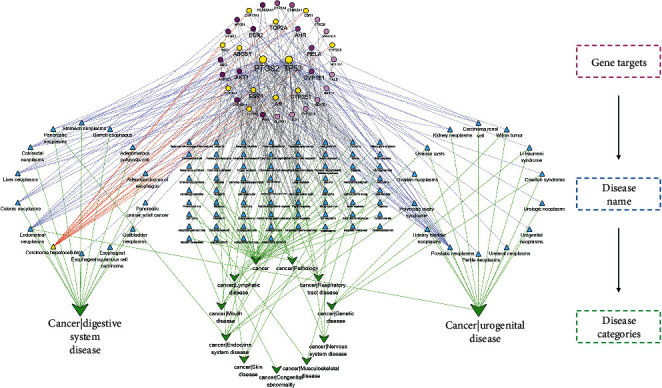
Antitumor location network in compound banmao capsule (CBC) against tumors. Round, triangle, and quadrilateral shapes stand for target, disease name, and disease categories. Yellow nodes stand for targets in hepatocellular carcinoma.

Based on Pharmacopoeia of the People's Republic of China, the 11 herbs of CBC were categorized according to the Four Properties and Five Flavors and imported into Cytoscape (Figure 6). Based on topological analysis, the pungent (degree = 5), bitter (degree = 5), and sweet (degree = 4) presented a higher degree value in nature and flavor. In addition, the liver-meridian (degree = 7), kidney-meridian (degree = 6), and spleen-meridian (degree = 6) had a higher degree value in channel tropism.

**Figure 7:**
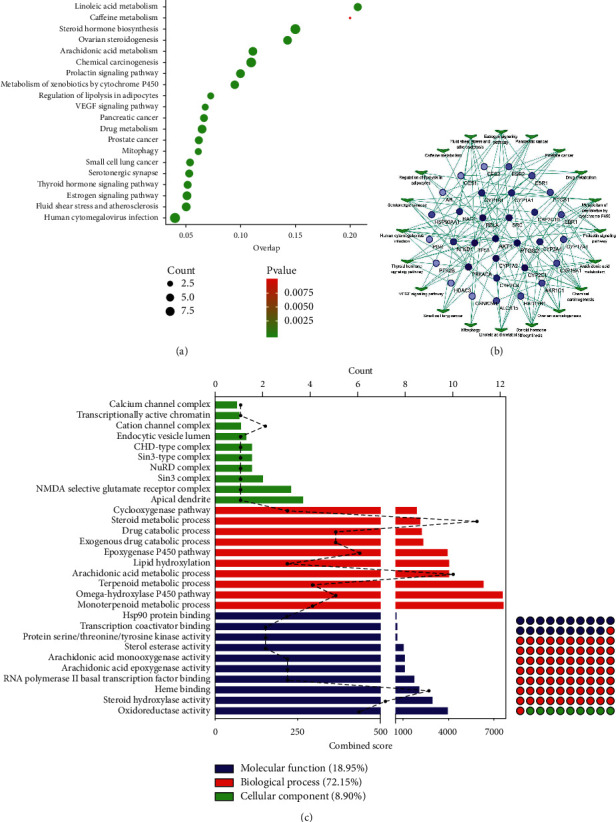
Enrichment analysis of targets in compound banmao capsule (CBC) against tumors. (a) Bubble plot of KEGG pathway analysis in CBC against tumors. *X* axis and *Y* axis stand for overlap and pathways respectively. (b) Target-pathway network in CBC. Round shape stands for count target; quadrilateral stands for pathway. Colors varying from shallow to deep indicate that the target degree value is increasing. (c) GO functional enrichment analysis in CBC against tumors. Bottom *X* axis, top *X* axis, and *Y* axis stand for combined score, count gene, and GO term. Green, red, and blue stand for cellular component (CC), biological processes (BP), and molecular function (MF), respectively.

A total of 96 diseases containing 13 categories of tumors were collected by enrichment analysis of 39 hub targets. Detail mapping relations were visualized via Cytoscape (Figure 7). Among them, the targets TP53 (degree = 58), PTGS2 (degree = 40), and ESR1 (degree = 20) showed a higher degree value in tumor location. Fourteen types of diseases were classified as cancer of digestive and urogenital systems. Compared with other diseases, breast neoplasms (degree = 21), prostatic neoplasms (degree = 19), and hepatocellular carcinoma (degree = 14) presented higher degree values. In general, the antitumor activity of CBC may have a superior efficacy in liver, kidney, and breast tumors.

#### 3.1.5. GO and Pathway Enrichment Analysis

Thirty-nine hub targets were imported into Enrichr database enrichment analysis. The top 20 KEGG pathways (*P* < 0.05) were visualized by bubble plot (Figure 7(a)), and target-pathway network was built using Cytoscape, obtaining 32 nodes of targets (Figure 7(b)). The top 10 GO enrichment analyses were performed individually (*P* < 0.05), including cellular component (CC), molecular function (MF), and biological processes (BP), which were ranked by a combined score (Figure 7(c)).

In this study, 132 pathways were enriched (*P* < 0.05), suggesting that the putative targets were highly connected with the regulation of prostate cancer, pancreatic cancer, estrogen signaling pathway, vascular endothelial growth factor (VEGF) signaling pathway, small cell lung cancer, and mitophagy. Alternatively, 290 GO terms were obtained (*P* < 0.05), including estrogen metabolic process, prostaglandin metabolic process, cellular response to angiotensin, estrogen biosynthetic process, and prostaglandin biosynthetic process, suggesting that CBC may influence cancer-related pathways, mitophagy, angiogenesis, and metabolism of estrogen and prostaglandin for tumor treatment.

#### 3.1.6. Multivariate Biological Network Build and Verification Analysis

**Figure 8:**
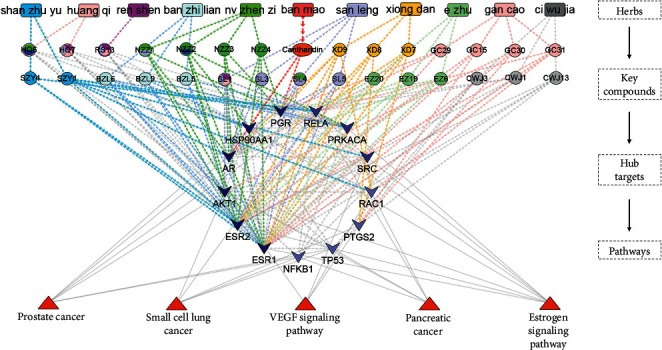
Herb-compound-target-pathway network of compound banmao capsule (CBC) against tumors. From top to bottom stand for herbs, key compounds, hub targets, and pathways, respectively. Nodes and edges of compounds come from the same herb and are defined with the same color. Colors varying from shallow to deep indicate that the target degree value is increasing in hub targets.

**Figure 9:**
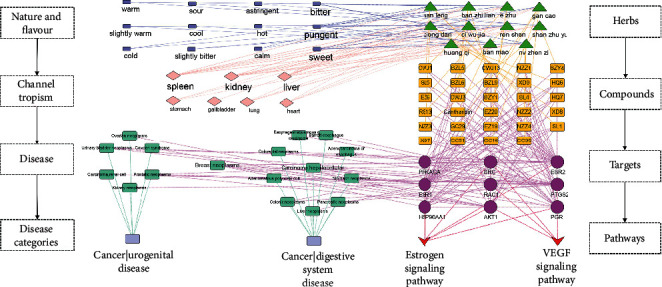
Multivariate-biological-network of compound banmao capsule (CBC). The left side stands for nature and flavor, channel tropism, and disease and disease categories, while the right side stands for herbs, compounds, targets, and pathways.

The herb-compound-target-pathway network was constructed using the target-pathway network and core compound-target network (Figure 8). The integrative network comprised 11 herbs, 26 key compounds, 13 hub targets, and five pathways, suggesting that the characteristics of multicompound-target-pathway of CBC, and each herbal drug was indispensable in tumor treatment. Among them, VEGF signaling pathway, estrogen signaling pathway, small cell lung cancer, pancreatic cancer, and prostate cancer were selected as mainly tumor-related pathways. Further integration of the multivariate biological network was merged based on VEGF and estrogen signaling pathways, involving nine targets, which are linked with 10 digestive system tumors, five urogenital tumors, and breast neoplasms (Figure 9).

**Figure 10:**
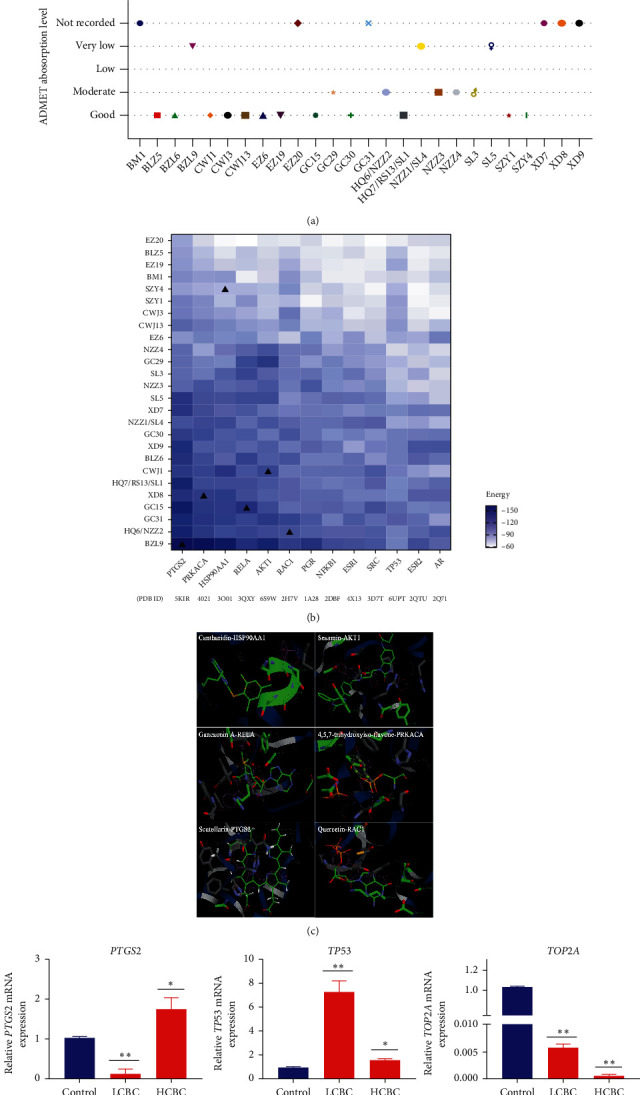
Results of predicted verification between compounds and targets in compound banmao capsule (CBC) against tumors. (a) Diagram of ADMET absorption level classification ratio of 26 key compounds of herbs from CBC. (b) Energy results in molecular docking. *X* axis and *Y* axis stand for gene targets and compounds. Colors varying from shallow to deep indicate that the docking accuracy is increasing. ▲ stands for displayed diagram in c. (c) Best pose of molecular docking in key compounds and targets. (d) Gene mRNA expression levels of top targets in hepatocellular carcinoma by quantitative real-time PCR (qPCR) of CBC in SMMC-7721 cells. Compared with control, ^*∗*^ presents *P* < 0.05 and ^*∗∗*^ presents *P* < 1.

To verify the accuracy of our prediction, 26 core compounds were examined by ADMET absorption level, suggesting that 65% of the compounds presented a good or moderate level (Figure 10(a)). Among them, 46% of the compounds showed a good level, indicating our prediction achieved great accuracy. Subsequently, 13 hub targets were docked with core compounds. The targets PTGS2 (−109.75), HSP90AA1 (−98.21), and AKT1 (−96.38) presented the most stable structures (Figure 10(b)). Gancaonin A (−108.53), quercetin (-108.80), scutellarin (−122.72), and sesamin (−102.37) showed a great potential against tumor targets by molecular docking and may be the main effective material basis of CBC. The ADMET absorption level of scutellarin was very low, but it still showed a great stable structure (Figure 10(c)).

#### 3.1.7. Results of qPCR for Liver Tumor Target Genes

The top five target genes *PTGS2*, *TP53*, *ESR1*, *ABCB1,* and *TOP2A* with a high degree of connectivity were identified in hepatocellular carcinoma of the antitumor location network. The effect of CBC on the expression of mRNA in SMMC-7721 cells was explored by qPCR (Figure 10(d)). In this study, the mRNA expression levels of *PTGS2*, *TOP2A*, *ESR1,* and *ABCB1* were decreased in the LCBC group (*P* < 0.01), while *PTGS2* was increased in the HCBC group (*P* < 0.05), and TP53 showed to be significantly increased in both doses (*P* < 0.01). Our results indicate that *PTGS2*, *TP53*, *ESR1*, *ABCB1,* and *TOP2A* may be the potential targets of CBC in the treatment of liver tumors.

## 4. Discussion

CBC is a traditional antitumor drug of Miao ethnic in Guizhou Province of China, although their effective material basis and mechanisms of action remain unclear [37]. In this study, CBC's bioactive compounds and antitumor targets were identified. A PPI network was constructed to calculate hub targets, which were enriched to explore potential synergistic effects of CBC's components. Finally, key compounds and targets were validated using molecular docking and qPCR.

CBC is clinically used as blood-activating, stasis-resolving, and detoxicating in TCM. Based on the Pharmacopoeia of the People's Republic of China, the 11 herbs of CBC were categorized according to the five properties and five flavors, reflecting the body's effect of “inclination” of the ups and downs of Yin and Yang and the “semi-interior phase” change during drug intervention [38]. To further explore the drug efficacy of complex compounds, nature and flavour analysis was applied. Our results indicated that CBC belonged to the pungent, bitter, and sweet categories. Pungent compounds have the potential action of “blood-activating” and “stasis-resolving” and may have potential in treating liver diseases [39]. Herbs with bitter taste can be used for “discharging” and “down-bearing,” referred to as “heat-clearing” and “detoxicating” herbs. Herbs with sweet taste may be used for “supplementation, moderation, and harmonization,” referred to as “tonifying and replenishing” herbs [40]. However, sweet-tasting herbs with “spleen-strengthening” functions are used more frequently than herbs with a bitter taste for “clearing heat.” There is a close relationship between recurrence and metastasis of breast cancer and liver. Herbs for “nourishing the yin-blood,” “emolliating and soothing the liver,” and “smoothing the meridians” are key in the treatment of breast cancer [40]. Our results indicated that CBC may have a multiaction “blood-activating,” “stasis-resolving,” “heat-clearing,” and “detoxicating,” and thus, it is potentially useful against breast and liver cancers.

To further explore the synergistic effects of CBC, the identification of the effective material basis and molecular mechanisms of CBC against tumors was analyzed.

### 4.1. Effective Material Basis of CBC

Twenty-six active compounds from 11 herbs were filtered as material basis of CBC by the herb-compound-target-pathway network analysis. In addition, the core compounds were verified by ADMET absorption level. Our results indicated that 65% of the core compounds showed a great absorption level, suggesting that our previous prediction was accurate. Among them, CTD, the core material basis of CBC and the most critical component in banmao, can efficiently inhibit the activity of mammalian and plant protein phosphatase 2A (PP2A) in the treatment of tumors [41], and CTD may elevate leukocyte and enhance immunity [42].

Similarly, other compounds with great absorption levels, such as baicalein, sesamin, quercetin, and kaempferol, indicated a better interaction with hub targets by molecular docking. Besides, scutellarin may positively interact with the studied targets. Flavonoids of BZL, including scutellarin and baicalein from CBC, have been found to significantly treat liver tumors [43, 44]. The antitumor effects of sesamin have been mainly attributed to apoptotic, inflammatory, metastatic, and autophagocytic activities [45, 46]. Quercetin exhibits anticancer properties, especially in cancers located in the digestive and urogenital systems [47, 48]. Kaempferol was able to treat various tumors in the liver, skin, and colon through apoptosis, cell cycle arrest, and epithelial-mesenchymal transition [49]. In summary, baicalein, scutellarin, sesamin, quercetin, and kaempferol may be the core components of CBC, which could influence the apoptotic, inflammatory, metastatic, and autophagic activity in tumor cells.

### 4.2. Antitumor Location of CBC and qPCR Verification of Liver Tumor Targets

The “channel tropism” is the core characteristic of TCM herbal property theory, which classifies drugs acting in the “body's viscera and meridians” to illustrate the selectivity of the drug's effects on a certain part of the body, thereby providing a basis for clinical dialectical medication [50]. Our results indicated that CBC belonged to “liver-meridian” and “kidney-meridian,” confirming its indications against tumors in the digestive and urogenital systems, based on TCM theories. Based on the antitumor location network analysis of CBC, digestive and urogenital systems tumors were mainly enriched in disease categories, especially in liver and breast cancer in disease terms.

The hub targets associated with hepatocellular carcinoma in antitumor location network of CBC were further verified by qPCR. The mRNA expression of *PTGS2*, *TP53*, *TOP2A, ESR1,* and *ABCB1* was significantly changed by CBC in SMMC-7721 cells, suggesting that these hub targets were crucial for the activity of CBC on hepatocellular carcinoma. The mRNA expression level of *PTGS2* indicated a contrary trend in both doses, suggesting a dynamic regulation in the treatment of tumors with CBC.

The mechanistic studies implicate PTGS2 is overexpressed in liver cancer for promoting angiogenesis and inflammation [51]. Studies have shown that EZ from CBC was able to inhibit hepatocellular carcinoma by inhibiting PTGS2 in SMMC-7721 cell [52]. TP53, a tumor suppressor gene, mediates DNA repair, apoptosis, cell cycle arrest, autophagy, and metabolic processes [53]. TP53 can be both an activator or inhibitor of autophagy [54]. Studies have shown that CTD may treat tumors by upregulating TP53 [55]. CTD was also able to inhibit breast cancer cells by suppressing autophagy and inducing apoptosis [56]. In addition, ESR1 may play a role in the development and prognosis of liver cancer [57]. The ABCB1 are considered to be a prime factor for inducting multidrug resistance in liver cancer treatment [58]. However, the effect of CBC on ESR1, TOP2A, and ABCB1 remains unclear. In this study, we concluded that CBC may dynamically regulate PTGS2, TP53, TOP2A, ESR1, and ABCB1 targets by inhibiting SMMC-7721 cells.

### 4.3. Integrative Biological Network Analysis

The herb-compound-target-pathway network indicated that potential mechanisms of CBC involved 13 targets and five pathways, including VEGF signaling pathway, estrogen signaling pathway, and three tumor-associated pathways. Of these 13 targets, AKT1, SRC, RAC1, and PTGS2 are involved in VEGF signaling pathway. VEGF can increase angiogenesis and vascular permeability, contributing to key aspects of tumorigenesis, including the function of cancer cells and tumor initiation [59]. More importantly, several herbs or compounds were able to inhibit the VEGF signaling pathway against tumors. CTD inhibited angiogenesis by downregulating VEGF in *vivo* and *vitro* [60], whereas astragaloside IV of HQ from CBC treated a glioma by inhibiting VEGF [61]. SZY from CBC treated osteosarcoma by downregulation of VEGF [62], and EZ and HQ from CBC activity against lung tumors were based on the inhibition of VEGF [63]. Our results indicated that the VEGF signaling pathway may be a critical pathway of the CBC's inhibition of angiogenesis.

AKT1, also known as protein kinase B, activates the PI3K/AKT pathway during tumor growth [64]. Studies have shown that targeting AKT1 may impair angiogenesis [65]. BM and EZ from CBC may inhibit AKT1 [66, 67]. In addition, increased SRC target activity was identified in several malignancies [68]. Activation of SRC may further stimulate PI3K/Akt pathway. Studies have shown that CTD and SZY from CBC may inhibit SRC in *vivo* and *vitro* [69, 70], but the regulation of SRC of CBC still remains unclear. Furthermore, the overexpression of PTGS2 was able to activate VEGF for tumor growth and diffusion [71], and EZ from CBC could treat liver cancer by inhibiting PTGS2 and VEGF [52]. Thus, PTGS2 may be a potential target of CBC.

Of 13 targets, AKT1, ESR1, ESR2, HSP90AA1, PGR, PRKACA, and SRC are involved in estrogen signaling pathway, and AKT1 and SRC both involved in both VEGF and estrogen signaling pathways. Sustained estrogen exposure or deregulated estrogen signaling is associated with various types of tumor, including breast, endometrium, and prostate. Estrogen is a potential mediator of immunosuppression through modulation of protumors [72]. Studies have shown that san leng from CBC may treat breast cancer by regulating the estrogen receptor [73]. EZ from CBC may treat breast cancer through the inhibition of estrogenic activity [74, 75].

The estrogen receptor signaling is mediated by estrogen receptors, ESR1 and ESR2, while the majority of breast cancers have disregulated estrogen receptor signaling [76]. ESR1 is able to mediate the biological effects of the steroid hormone estrogen, while its mutation in breast cancer is a common mechanism of hormonal therapy resistance [77]. With the inhibition of ESR1, the risk of hormone breast cancer would be diminished [78]. ESR2 has been used as a prognostic molecular biomarker [79].

In this study, effective material basis, antitumor location, and mechanism of CBC were integrated in a multivariate biological network (Figure 9), which combined TCM theories and molecular biological network, identifying the multicompound-target-pathway of CBC against tumors. Our results indicated that CBC displays is “blood-activating,” “stasis-resolving,” “heat-clearing,” and “detoxicating,” and this is associated with its pharmacological function in VEGF, estrogen signaling pathway, PTGS2, and ESR1. These targets were further verified by qPCR. Furthermore, CBC's activity as antitumor against liver and breast tumors was confirmed by a literature review.

## 5. Conclusion

Effective material basis and potential molecular mechanisms of CBC against tumors have been here explored, based on TCM theories and biological network analysis. These were further verified by qPCR and molecular docking. The potential therapeutic location and hub targets of CBC were identified by multivariate biological network integration. Future studies should focus on CBC's mechanisms of action by molecular experiments in vivo and in vitro. Additionally, these experiments would further validate our results as per the inherent limitations of network predictions.

## Figures and Tables

**Table 1:** Primers used for quantitative real-time PCR (qPCR).

Gene	Forward primer	Reverse primer
*PTGS2*	CTGGCGCTCAGCCATACAG	CGCACTTATACTGGTCAAATCCC
*TP53*	GAGGTTGGCTCTGACTGTACC	TCCGTCCCAGTAGATTACCAC
*ESR1*	GAAAGGTGGGATACGAAAAGACC	GCTGTTCTTCTTAGAGCGTTTGA
*ABCB1*	GGGATGGTCAGTGTTGATGGA	GCTATCGTGGTGGCAAACAATA
*TOP2A*	ACCATTGCAGCCTGTAAATGA	GGGCGGAGCAAAATATGTTCC
*GAPDH*	CTGGGCTACACTGAGCACC	AAGTGGTCGTTGAGGGCAATG

Abbreviations. qPCR: quantitative real-time PCR; PTGS2: prostaglandin G/H synthase 2; TP53: cellular tumor antigen p53; ESR1: estrogen receptor; ABCB1: ATP-dependent translocase ABCB1; TOP2A: DNA topoisomerase 2-alpha.

**Table 2:** Active compounds of compound banmao capsule (CBC).

Herb	No.	Compound
Mylabris (banmao)	BM1	Cantharidin
Ginseng Radix Et Rhizoma (ren shen)	RS1	Gomisin A
	RS2	Vitamin B1
	RS3	Butylated hydroxytoluene
	RS4	Α-Santalol
	RS5	Deoxygomisin A
	RS6	2,5-Dimethyl-7-hydroxy chromone
	RS7	Ginsenoside rc
	RS8	Ginsenoside rd
	RS9	Ginsenoside Re
	RS10	Ginsenoside Rg3
	RS11	Ginsenoside Rh4
	RS12	Ginsenoside Rh2
	RS13	Kaempferol
	RS14	Ginsenoside Rb1
	RS15	Ginsenoside Rg1
Acanthopanacis Senticosi Radix Et Rhizoma Seu Caulis (ci wu jia)	CWJ1	Sesamin
	CWJ2	Neociwujiaphenol
	CWJ3	Syringic acid
	CWJ4	Syringaresinol
	CWJ5	3-O-*trans*-ferulylquinic acid
	CWJ6	Hederasaponin B
	CWJ7	Eleutheroside K
	CWJ8	Ciwujianoside C1
	CWJ9	Ciwujianoside D1
	CWJ10	Ciwujianoside B
	CWJ11	Ciwujianoside D2
	CWJ12	Syringin
	CWJ13	Isofraxidin
Curcumae Rhizoma (e zhu)	EZ1	7-Hydroxy-5-methoxyflavanone
	EZ2	Pinocembrin
	EZ3	Curcarabranol A
	EZ4	(1S,3 R,6R,7 R)-1-Methyl-7-(2-(2-methyl-1,3-dioxolan-2-Yl) ethyl)-4-(propan-2-ylidene) bicyclo [4.1.0] Heptan-3-ol
	EZ5	(4Ar,5R,5 As,6Ar)-6a-Hydroxy-3,5a-dimethyl-5-(3-oxobutyl)-4,4A,5,5A,6,6A-hexahydro-2h-cyclopropa (F) [1] benzofuran-2-one
	EZ6	(5S,8 R,9S,10S,13S,14S)-3-Ethyl-3-hydroxy-10,13-dimethyl-tetradecahydro-2h-cyclopenta [A] phenanthren-17 (14H)-one
	EZ7	(8S,8 As)-8-Hydroxy-3,5,8a-trimethyl-7,8,8A,9-tetrahydronaphtho [2,3 B] furan-4 (6H)-one
	EZ8	Dihydrocurcumenone
	EZ9	4-((1S,6 R,7R)-4-(2-Hydroxypropan-2-yl)-1-methylbicyclo [4.1.0] hept-3-en-7-Yl) butan-2-one
	EZ10	(4Ar,5R,5 As,6 As)-3,5a-Dimethyl-5-(3-oxobutyl)-4,4A,5,5A,6,6A-hexahydro-2h-cyclopropa (F) [1] benzofuran-2-one
	EZ11	Epicurzerenone
	EZ12	Curzerenone
	EZ13	Zedoarol
	EZ14	(3S,3 As,5S,8 As)-3a-Hydroxy-3,3′,3′,8-tetramethyl-1,2,3,3A,4,8A-hexahydro-6h-spiro [azulene-5,2′-oxiran]-6-one
	EZ15	Curcumenone
	EZ16	(S)-2-Methyl-6-(4-methylcyclohex-3-en-1-Yl) hepta-2,6-dien-1-ol
	EZ17	Ar-Turmerone
	EZ18	Isovelleral
	EZ19	Azulen-5-ylmethanol
	EZ20	*β*-Elemene
	EZ21	Curcumol
	EZ22	Curdione
Ligustri Lucidi Fructus (nv zhen zi)	NZZ1	*β*-Sitosterol
	NZZ2	Quercetin
	NZZ3	Ursolic acid
	NZZ4	Oleanolic acid
	NZZ5	Specnuezhenide
	NZZ6	Ligustroflavone
Fel Ursi (xiong dan)	XD1	Ursodeoxycholic acid
	XD2	Tauroursodeoxycholic acid
	XD3	Deoxycholic acid
	XD4	Cholic acid
	XD5	Taurochenodeoxycholic acid
	XD6	Chenodeoxycholic acid
	XD7	4,7-Dihydroxyisoflavone
	XD8	4,5,7-Trihydroxyiso-flavone
	XD9	4,7-Dihydroxy-6-methoxyflavone
Glycyrrhizae Radix Et Rhizoma (gan cao)	GC32	Glabridin
	GC33	Licoricidin
Astragali Radix (huang qi)	HQ1	Kumatakenin
	HQ2	Medicarpin
	HQ3	Isorhamnetin
	HQ4	Calycosin
	HQ5	Formononetin
	HQ6	Quercetin
	HQ7	Kaempferol
	HQ8	7-O-Methylisomucronulatol
	HQ9	Astragaloside iv
	HQ10	Astragaloside II
	HQ11	Astramembrannin ii
Sparganii Rhizoma (san leng)	SL1	Kaempferol
	SL2	Formononetin
	SL3	Hederagenin
	SL4	*β*-Sitosterol
	SL5	Stigmasterol
	SL6	Trans-gondoic acid
Scutellariae Barbatae Herba (ban zhi lian)	BZL1	7-Hydroxy-5,8-dimethoxyflavone
	BZL2	Wogonin
	BZL3	Rivularin
	BZL4	4′-Hydroxywogonin,5,7-dihydroxy-8-methoxylflavone
	BZL5	*p-*Hydroxybenzyl acetone
	BZL6	Baicalein
	BZL7	Scutebarbatines A
	BZL8	Scutebarbatines B
	BZL9	Scutellarin
Corni Fructus (shan zhu yu)	SZY1	Isoasarone
	SZY2	Elemicin
	SZY3	1-Allyl-2,4,5-trimethoxy-benzene
	SZY4	Ethylvanillin
	SZY5	Asaricin
	SZY6	Retinol
	SZY7	Iridoid
	SZY8	Loganin
	SZY9	Morroniside
Glycyrrhizae Radix Et Rhizoma (gan cao)	GC1	Formononetin
	GC2	3′,7-Dihydroxy-4′,6-dimethoxyisoflavone
	GC3	Kumatakenin
	GC4	Gancaonin X
	GC5	4′-O-Methylglabridin
	GC6	Gancaonin Y
	GC7	3′-Methoxyglabridin
	GC8	Glycyrrhisoflavanone
	GC9	Glyzaglabrin
	GC10	Phaseollinisoflavan
	GC11	(S)-5,7-Dihydroxy-2-phenylchroman-4-one, pinocembrin
	GC12	Liquiritigenin
	GC13	Licoagroisoflavone
	GC14	Licoagropin
	GC15	Gancaonin A
	GC16	Maackiain
	GC17	3,3′-Dimethylquercetin
	GC18	Erythrinin C
	GC19	Gancaonin Z
	GC20	Licoricone
	GC21	Hispaglabridin B
	GC22	Lupiwighteone
	GC23	Semilicoisoflavone B
	GC24	Licoisoflavone B
	GC25	Xambioona
	GC26	Gancaonin B
	GC27	Glicoricone
	GC28	Glycyrrhizic acid
	GC29	Glycyrrhetinic acid
	GC30	Isoliquiritigenin
	GC31	Licochalcone A
	GC34	Isoangustone A
	GC35	Liquiritin

Abbreviations. QED: quantitative estimate of drug-likeness; BM: Mylabris; RS: Ginseng Radix Et Rhizoma; HQ: Astragali Radix; CWJ: Acanthopanacis Senticosi Radix Et Rhizoma Seu Caulis; SL: Sparganii Rhizoma; EZ: Curcumae Rhizoma; BZL: Scutellariae Barbatae Herba; SZY: Corni Fructus; NZZ: Ligustri Lucidi Fructus; XD: Fel Ursi; GC: Glycyrrhizae Radix Et Rhizoma.

## Data Availability

The data used to support the findings of this study are included within the article.

## Supplementary Materials

Supplementary Materials Table 1: detail compounds in each herb from compound banmao capsule (CBC).
