# Supplementary material for: Effective Material Basis and Mechanism Analysis of Compound Banmao Capsule against Tumors Using Integrative Network Pharmacology and Molecular Docking
Source: Evid Based Complement Alternat Med. 2021 May 4;2021:6653460. doi: 10.1155/2021/6653460 (PMC8112962; doi:10.1155/2021/6653460)
Supplement: Supplementary Materials — Table 1: detail compounds in each herb from compound banmao capsule (CBC). [file 6653460.f1.docx]

**Supplement material**

**Table1 Detail compounds in each herb from compound banmao capsule(CBC)**

| **Herb** | **No.** | **Compound name** | **QED** | **Source** |
| --- | --- | --- | --- | --- |
| *Mylabris phalerata* Pallas  (1.56%,Mylabris, ban mao, BM) | BM1 | Cantharidin | - | Reports |
| Panax ginseng C. A. Mey  (3.90%, Ginseng Radix Et Rhizoma, ren shen, RS) | RS1 | Gomisin A | 0.811 | ETCM |
|  | RS2 | Vitamin B1 | 0.806 | ETCM |
|  | RS3 | Butylated hydroxytoluene | 0.753 | ETCM |
|  | RS4 | Α-Santalol | 0.722 | ETCM |
|  | RS5 | Deoxygomisin A | 0.714 | ETCM |
|  | RS6 | 2,5-Dimethyl-7-Hydroxy Chromone | 0.681 | ETCM |
|  | RS7 | Ginsenoside Rc | 0.067 | Reports |
|  | RS8 | Ginsenoside Rd | 0.089 | Reports |
|  | RS9 | Ginsenoside Re | 0.092 | Reports |
|  | RS10 | Ginsenoside Rg3 | 0.115 | Reports |
|  | RS11 | Ginsenoside Rh4 | 0.195 | Reports |
|  | RS12 | Ginsenoside Rh2 | 0.185 | Reports |
|  | RS13 | Kaempferol | 0.637 | Reports |
|  | RS14 | Ginsenoside Rb1 | 0.064 | Reports |
|  | RS15 | Ginsenoside Rg1 | 0.11 | Reports |
| Astragalus membranaceus (Fisch.) Bge  (19.51%, Astragali Radix, huang qi, HQ) | HQ1 | Kumatakenin | 0.904 | ETCM |
|  | HQ2 | Medicarpin | 0.865 | ETCM |
|  | HQ3 | Isorhamnetin | 0.668 | Reports |
|  | HQ4 | Calycosin | - | Reports |
|  | HQ5 | Formononetin | 0.909 | ETCM |
|  | HQ6 | Quercetin | 0.506 | Reports |
|  | HQ7 | Kaempferol | 0.637 | Reports |
|  | HQ8 | 7-O-methylisomucronulatol | - | Reports |
|  | HQ9 | Astragaloside Iv | 0.151 | Reports |
|  | HQ10 | Astragaloside II | - | Reports |
|  | HQ11 | Astramembrannin Ii | 0.256 | Reports |
| Acanthopanax senticosus (Rupr. et Maxim.) Harms  (19.51%, Acanthopanacis Senticosi Radix Et Rhizoma Seu Caulis,  ci wu jia, CWJ) | CWJ1 | Sesamin | 0.824 | ETCM |
|  | CWJ2 | Neociwujiaphenol | 0.795 | ETCM |
|  | CWJ3 | Syringic Acid | 0.762 | ETCM |
|  | CWJ4 | Syringaresinol | 0.737 | ETCM |
|  | CWJ5 | 3-O-trans ferulylquinic acid | 0.718 | ETCM |
|  | CWJ6 | Hederasaponin B | - | Reports |
|  | CWJ7 | Eleutheroside K | 0.175 | Reports |
|  | CWJ8 | Ciwujianoside C1 | 0.092 | Reports |
|  | CWJ9 | Ciwujianoside D1 | 0.086 | Reports |
|  | CWJ10 | Ciwujianoside B | 0.075 | Reports |
|  | CWJ11 | Ciwujianoside D2 | 0.07 | Reports |
|  | CWJ12 | Syringin | 0.428 | Reports |
|  | CWJ13 | Isofraxidin | 0.608 | Reports |
| *Sparganium stoloniferum* Buch. -Ham  (6.23%, Sparganii Rhizoma, san leng, SL) | SL1 | Kaempferol | 0.637 | Reports |
|  | SL2 | Formononetin | 0.909 | ETCM |
|  | SL3 | Hederagenin | 0.473 | Reports |
|  | SL4 | β-sitosterol | 0.435 | Reports |
|  | SL5 | Stigmasterol | 0.46 | Reports |
|  | SL6 | Trans-gondoic acid | - | Reports |
| Curcuma phaeocaulis VaL  (6.23%, Curcumae Rhizoma, e zhu, EZ) | EZ1 | 7-Hydroxy-5-Methoxyflavanone | 0.911 | ETCM |
|  | EZ2 | Pinocembrin | 0.823 | ETCM |
|  | EZ3 | Curcarabranol A | 0.816 | ETCM |
|  | EZ4 | (1S,3R,6R,7R)-1-Methyl-7-(2-(2-Methyl-1,3-Dioxolan-2-Yl)Ethyl)-4-(Propan-2-Ylidene)Bicyclo[4.1.0]Heptan-3-Ol | 0.807 | ETCM |
|  | EZ5 | (4Ar,5R,5As,6Ar)-6A-Hydroxy-3,5A-Dimethyl-5-(3-Oxobutyl)-4,4A,5,5A,6,6A-Hexahydro-2H-Cyclopropa[F][1]Benzofuran-2-One | 0.784 | ETCM |
|  | EZ6 | (5S,8R,9S,10S,13S,14S)-3-Ethyl-3-Hydroxy-10,13-Dimethyl-Tetradecahydro-2H-Cyclopenta[A]Phenanthren-17(14H)-One | 0.782 | ETCM |
|  | EZ7 | (8S,8As)-8-Hydroxy-3,5,8A-Trimethyl-7,8,8A,9-Tetrahydronaphtho[2,3-B]Furan-4(6H)-One | 0.765 | ETCM |
|  | EZ8 | Dihydrocurcumenone | 0.765 | ETCM |
|  | EZ9 | 4-((1S,6R,7R)-4-(2-Hydroxypropan-2-Yl)-1-Methylbicyclo[4.1.0]Hept-3-En-7-Yl)Butan-2-One | 0.759 | ETCM |
|  | EZ10 | (4Ar,5R,5As,6As)-3,5A-Dimethyl-5-(3-Oxobutyl)-4,4A,5,5A,6,6A-Hexahydro-2H-Cyclopropa[F][1]Benzofuran-2-One | 0.719 | ETCM |
|  | EZ11 | Epicurzerenone | 0.717 | ETCM |
|  | EZ12 | Curzerenone | 0.717 | ETCM |
|  | EZ13 | Zedoarol | 0.715 | ETCM |
|  | EZ14 | (3S,3As,5S,8As)-3A-Hydroxy-3,3',3',8-Tetramethyl-1,2,3,3A,4,8A-Hexahydro-6H-Spiro[Azulene-5,2'-Oxiran]-6-One | 0.713 | ETCM |
|  | EZ15 | Curcumenone | 0.703 | ETCM |
|  | EZ16 | (S)-2-Methyl-6-(4-Methylcyclohex-3-En-1-Yl)Hepta-2,6-Dien-1-Ol | 0.687 | ETCM |
|  | EZ17 | Ar-Turmerone | 0.684 | ETCM |
|  | EZ18 | Isovelleral | 0.684 | ETCM |
|  | EZ19 | Azulen-5-Ylmethanol | 0.674 | ETCM |
|  | EZ20 | β-elemene | - | Reports |
|  | EZ21 | Curcumol | 0.709 | ETCM |
|  | EZ22 | Curdione | 0.654 | Reports |
| Scutellaria barbata D. Don  (23.41%, Scutellariae Barbatae Herba, ban zhi lian, BZL) | BZL1 | 7-Hydroxy-5,8-Dimethoxyflavone | 0.944 | ETCM |
|  | BZL2 | Wogonin | 0.886 | ETCM |
|  | BZL3 | Rivularin | 0.88 | ETCM |
|  | BZL4 | 4'-Hydroxywogonin,5,7-Dihydroxy-8-Methoxylflavone | 0.788 | ETCM |
|  | BZL5 | P-Hydroxybenzyl Acetone | 0.691 | ETCM |
|  | BZL6 | Baicalein | 0.693 | ETCM |
|  | BZL7 | Scutebarbatines A | - | Reports |
|  | BZL8 | Scutebarbatines B | - | Reports |
|  | BZL9 | Scutellarin | 0.308 | Reports |
| *Cornus officinalis* Sieb. et Zucc  (7.80%, Corni Fructus, shan zhu yu, SZY) | SZY1 | Isoasarone | 0.761 | ETCM |
|  | SZY2 | Elemicin | 0.696 | ETCM |
|  | SZY3 | 1-Allyl-2,4,5-Trimethoxy-Benzene | 0.696 | ETCM |
|  | SZY4 | Ethylvanillin | 0.695 | ETCM |
|  | SZY5 | Asaricin | 0.689 | ETCM |
|  | SZY6 | Retinol | 0.678 | ETCM |
|  | SZY7 | Iridoid | - | Reports |
|  | SZY8 | Loganin | 0.336 | Reports |
|  | SZY9 | Morroniside | 0.312 | Reports |
| *Ligustrum lucidum* Ait  (7.80%, Ligustri Lucidi Fructus, nv zhen zi, NZZ) | NZZ1 | β-sitosterol | 0.435 | Reports |
|  | NZZ2 | Quercetin | 0.506 | Reports |
|  | NZZ3 | Ursolic Acid | 0.443 | Reports |
|  | NZZ4 | Oleanolic acid | 0.446 | Reports |
|  | NZZ5 | Specnuezhenide | - | Reports |
|  | NZZ6 | Ligustroflavone | - | Reports |
| *Selenarctos thibetanus* Cuvier  (0.16%, Fel Ursi, xiong dan, XD) | XD1 | Ursodeoxycholic acid | - | Reports |
|  | XD2 | Tauroursodeoxycholic acid | - | Reports |
|  | XD3 | Deoxycholic acid | - | Reports |
|  | XD4 | Cholic acid | - | Reports |
|  | XD5 | Taurochenodeoxycholic acid | - | Reports |
|  | XD6 | Chenodeoxycholic acid | - | Reports |
|  | XD7 | 4,7-dihydroxyisoflavone | - | Reports |
|  | XD8 | 4,5,7-trihydroxyiso-flavone | - | Reports |
|  | XD9 | 4,7-dihydroxy-6-methoxyflavone | - | Reports |
| *Glycyrrhiza uralensis* Fisch  (3.90%, Glycyrrhizae Radix et Rhizoma, gan cao, GC) | GC1 | Formononetin | 0.909 | ETCM |
|  | GC2 | 3',7-Dihydroxy-4',6-Dimethoxyisoflavone | 0.904 | ETCM |
|  | GC3 | Kumatakenin | 0.904 | ETCM |
|  | GC4 | Gancaonin X | 0.89 | ETCM |
|  | GC5 | 4'-O-Methylglabridin | 0.89 | ETCM |
|  | GC6 | Gancaonin Y | 0.89 | ETCM |
|  | GC7 | 3'-Methoxyglabridin | 0.854 | ETCM |
|  | GC8 | Glycyrrhisoflavanone | 0.846 | ETCM |
|  | GC9 | Glyzaglabrin | 0.839 | ETCM |
|  | GC10 | Phaseollinisoflavan | 0.832 | ETCM |
|  | GC11 | (S)-5,7-Dihydroxy-2-Phenylchroman-4-One,Pinocembrin | 0.823 | ETCM |
|  | GC12 | Liquiritigenin | 0.823 | ETCM |
|  | GC13 | Licoagroisoflavone | 0.821 | ETCM |
|  | GC14 | Licoagropin | 0.808 | ETCM |
|  | GC15 | Gancaonin A | 0.806 | ETCM |
|  | GC16 | Maackiain | 0.805 | ETCM |
|  | GC17 | 3,3'-Dimethylquercetin | 0.791 | ETCM |
|  | GC18 | Erythrinin C | 0.767 | ETCM |
|  | GC19 | Gancaonin Z | 0.765 | ETCM |
|  | GC20 | Licoricone | 0.754 | ETCM |
|  | GC21 | Hispaglabridin B | 0.742 | ETCM |
|  | GC22 | Lupiwighteone | 0.737 | ETCM |
|  | GC23 | Semilicoisoflavone B | 0.73 | ETCM |
|  | GC24 | Licoisoflavone B | 0.73 | ETCM |
|  | GC25 | Xambioona | 0.712 | ETCM |
|  | GC26 | Gancaonin B | 0.707 | ETCM |
|  | GC27 | Glicoricone | 0.707 | ETCM |
|  | GC28 | Glycyrrhizic Acid | 0.135 | Reports |
|  | GC29 | Glycyrrhetinic Acid | 0.519 | Reports |
|  | GC30 | Isoliquiritigenin | 0.582 | Reports |
|  | GC31 | Licochalcone A | - | Reports |
|  | GC32 | Glabridin | - | Reports |
|  | GC33 | Licoricidin | 0.452 | Reports |
|  | GC34 | Isoangustone A | - | Reports |
|  | GC35 | Liquiritin | 0.484 | Reports |
